# Evaluation of left ventricular function after percutaneous recanalization of chronic coronary occlusions: The role of two-dimensional speckle tracking echocardiography

**DOI:** 10.1007/s00059-017-4663-1

**Published:** 2018-01-16

**Authors:** P. Wang, Y. Liu, L. Ren

**Affiliations:** 1 Department of Cardiology, Beijing Shijitan Hospital, Capital Medical University, No. 10 Tieyi Road, 100038 Beijing, China; 2 Department of Ultrasound, Beijing Shijitan Hospital, Capital Medical University, No. 10 Tieyi Road, 100038 Beijing, China

**Keywords:** Coronary occlusion, Echocardiography, Percutaneous coronary intervention, Left ventricular ejection fraction, Myocardial infarction, Koronarverschluss, Echokardiographie, Perkutane Koronarintervention, Linksventrikuläre Ejektionsfraktion, Myokardinfarkt

## Abstract

**Background:**

This study evaluated the feasibility of using two-dimensional speckle tracking echocardiography (2D-STE) to monitor left ventricular (LV) and overall function after percutaneous recanalization.

**Methods:**

LV function after percutaneous recanalization was monitored by 2D-STE and conventional echocardiography in 43 patients with coronary chronic total occlusion (CTO) who underwent primary percutaneous coronary intervention (PCI). Follow-ups were carried out 1 day as well as 3 and 6 months after CTO-PCI. At each time point, LV ejection fraction (LVEF) was examined by echocardiography, and LV global longitudinal strain (GLS) was measured by 2D-STE.

**Results:**

It was found that the global longitudinal strain assessed with 2D-STE was improved as early as 1 day after CTO-PCI, whereas LVEF tended to improve up to 3 and 6 months after CTO-PCI.

**Conclusion:**

PCI can effectively improve LV function in patients with CTO. 2D-STE is a superior technique for objectively quantifying the functional change earlier.

## Zusammenfassung

**Hintergrund:**

Die vorliegende Studie befasst sich mit der Eignung der zweidimensionalen Speckle-Tracking-Echokardiographie (2D-STE) zur Überwachung der linksventrikulären (LV) und Gesamtfunktion nach perkutaner Rekanalisation.

**Methoden:**

Die LV-Funktion nach perkutaner Rekanalisation wurde bei 43 Patienten mit koronarer chronischer Totalokklusion („coronary chronic total occlusion“, CTO), bei denen eine primäre perkutane Koronarintervention („percutaneous coronary intervention“, PCI) erfolgt war, mittels 2D-STE und herkömmlicher Echokardiographie überwacht. Nachuntersuchungen wurden einen Tag sowie 3 und 6 Monate nach CTO-PCI durchgeführt. Zu jedem Zeitpunkt wurde die LV-Ejektionsfraktion (LVEF) mittels Echokardiographie und die LV-Gesamt-Longitudinaldeformation („global longitudinal strain“, GLS) mittels 2D-STE gemessen.

**Ergebnisse:**

Die Autoren stellten fest, dass die mit 2D-STE erfasste GLS sich schon einen Tag nach CTO-PCI verbessert hatte, während die LVEF sich 3 bzw. 6 Monaten nach CTP-PCI allmählich besserte.

**Schlussfolgerung:**

Eine PCI kann die LV-Funktion bei Patienten mit CTO wirksam verbessern. Die 2D-STE erwies sich als überlegenes Verfahren für die frühzeitige objektive quantitative Beurteilung funktioneller Veränderungen

Chronic total occlusion (CTO) is defined as an occluded coronary artery presenting as thrombolysis in myocardial infarction (TIMI) with grade 0 or 1 flow in a recent myocardial infarction (MI) [1, 2]. The myocardium in the perfusion territory of a CTO can be functional, dysfunctional but viable, or dysfunctional and nonviable [3, 4]. Dysfunctional but viable myocardium in CTO may have restored function after percutaneous coronary intervention (PCI) [5–9]. However, the earliest time that the recovery of dysfunctional but viable myocardium can be detected remains unknown. Conventional echocardiography enables us to identify significant left ventricular (LV) dysfunction but not subclinical dysfunction [10]. Two-dimensional speckle tracking echocardiography (2D-STE) allows for an angle-independent evaluation of myocardial strain, and provides comprehensive information on LV myocardial contractility. Thus, 2D-STE is superior in detecting subtle deteriorations of contractility [11–13]. These advantages of 2D-STE are useful for the detection of subclinical recovery of dysfunctional but viable myocardium after CTO-PCI.

In this study, the LV systolic function of CTO patients with dysfunctional myocardium was evaluated at 1 day as well as 3 and 6 months after percutaneous revascularization using 2D-STE. It was found that 2D-STE was superior in detecting subclinical LV systolic dysfunction in CTO patients after percutaneous revascularization.

## Patients and methods

### Subjects

A single-center prospective observational study was performed from July 2012 to March 2016. Patients with CTO with an estimated duration of less than 3 months or an MI during the previous 30 days were excluded, but no other predefined clinical inclusion or exclusion criteria were applied. The indication for PCI was determined by individual investigators at the participating center. In all, 43 patients (mean age: 66.09 ± 10.99 years) scheduled to undergo PCI for CTO in a native coronary artery were eligible for enrollment. Among them, there were 40 males and three females. The CTO of ten patients was located in the left anterior descending artery in ten patients, in the right coronary artery in 25 patients, and in the circumflex artery in eight patients. A follow-up visit or telephone interview was scheduled at 1 day as well as 3 and 6 months after percutaneous revascularization. Prior written and informed consent was obtained from every patient. This study was approved by the Ethics Review Board of Capital Medical University.

### Percutaneous revascularization

Percutaneous revascularization was performed by operators highly experienced in the treatment of CTO according to their standard practices with the femoral or brachial approach. The operation was considered successful when the residual stenosis was ≤30% of the intraluminal diameter, with TIMI grade 3 flow and no in-hospital complications (death, acute myocardial infarction, or emergency coronary surgery). Clopidogrel (75 mg daily) was prescribed to all patients for at least 1 year after stent implantations, and aspirin was given indefinitely.

### Basic echocardiographic measurement and 2D-STE

Echocardiography was performed using a Philips IE33 ultrasound machine (Koninklijke Philips N.V., The Netherlands) with an M4S transducer according to the recommendations of the American Society of Echocardiography [4, 13]. The percentage of the left ventricular ejection fraction (LVEF) was calculated by Simpson’s biplane method of discs.

The 2D echocardiography images (transmit/receive 1.9/4.0 MHz) were obtained from several views with frame rates of 30–90 frames/s. Digital data were stored and analyzed off-line. LV endocardial surface was traced manually, and the speckle tracking width was modified to cover the whole LV wall thickness so as to obtain curves for the peak longitudinal strain of: (a) the inferior septum and lateral wall in the apical four-chamber view (4C-PLS); (b) the inferior and anterior walls in the apical two-chamber view (2C-PLS); and (c) the inferior lateral and anterior septum in the apical three-chamber view (3C-PLS). LV global longitudinal systolic strain (GLS) was calculated by averaging the peak systolic values of the six LV walls. All the echocardiographic studies were performed by one echocardiographer. In terms of intraobserver variability, a sample of 2D strain was randomly selected and examined by the same observer in 2 days and the intraclass correlation coefficients were also calculated. All data were analyzed using Philips QLAB Advanced ultrasound quantification software (release 8.1).

### Statistical analysis

The collected data were analyzed using the Statistical Package for Social Science (SPSS, version 19.0). Differences among groups were compared using the two-tailed Student’s *t* test or chi-square analysis. Statistical significance was defined as a *p* value of less than 0.05.

## Results

All the images of the 43 patients were suitable for analysis. Improvement of LVEF by CTO-PCI was observed at up to 3 and 6 months. The clinical characteristics of the 43 patients are listed in Table 1. The LVEF and GLS variables of all 43 patients before recanalization and at the follow-ups are listed in Table 2.

**Table 1:** Patient characteristics

Variable	Value
Men	40
Mean age (years)	66.09 ± 10.99
LVEDD (mm)	46 ± 2
LVESD (mm)	29 ± 3
IVS thickness (mm)	10.7 ± 2.1
PW thickness (mm)	10.5 ± 2.3
LA diameter (mm)	42 ± 2
RA diameter (mm)	29 ± 3

*LVEDD* left ventricular end-diastolic dimension, *LVESD* left ventricular end-systolic dimension, *IVS* interventricular septum, *PW *post wall, *LA* left atrium, *RA* right atrium

**Table 2:** LVEF and GLS variables of the 43 patients before PCI and at follow-up

	LVEF	*p*	GLS	*p*
PRO	59.35 ± 10.16	–	−13.25 ± 1.86	–
1 Day	60.35 ± 10.48	0.112^a^	−14.54 ± 2.06	<0.001^a^
3 Months	61.95 ± 10.20	<0.001^b^	−15.51 ± 2.05	<0.001^b^
6 Months	65.86 ± 9.83	<0.001^c^	−16.58 ± 2.17	<0.001^c^

*LVEF* left ventricular ejection fraction, *PRO* preoperative, *PEF* preoperative ejection fraction, *GLS* global longitudinal strain, *PG* preoperative global longitudinal strain

^a^Preoperative vs. at 1 day

^b^Preoperative vs. at 3 months

^c^Preoperative vs. at 6 months

**Fig. 1:**
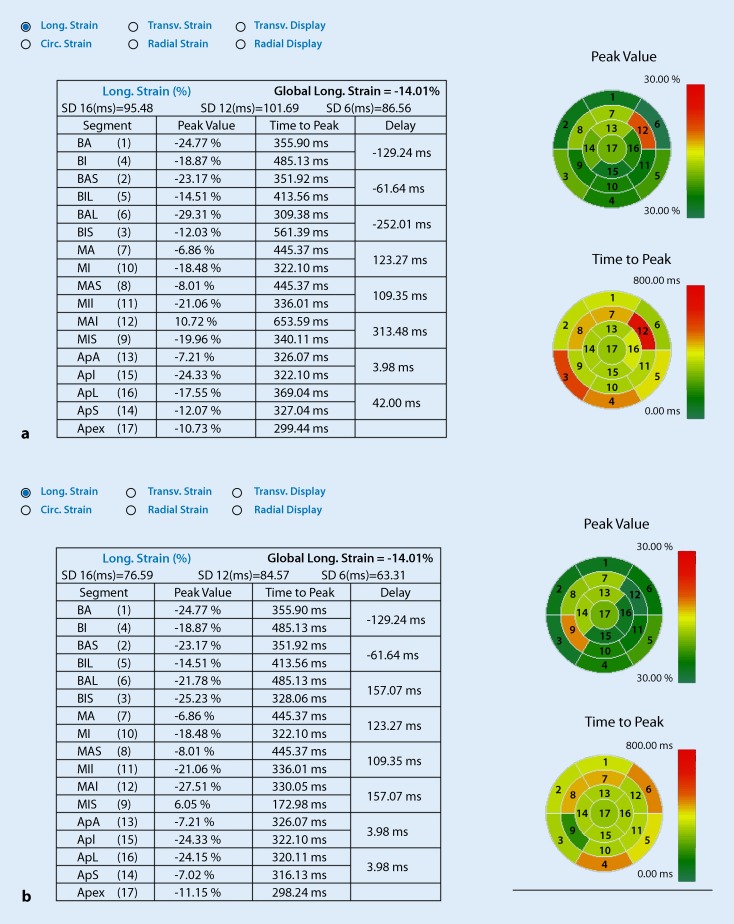
Significant improvement in global longitudinal strain (GLS) after percutaneous coronary intervention (PCI). **a** GLS = −14.01, 1 day before PCI. **b** GLS = −15.03, 1 day after PCI. *Circ*. circular, *Long*. longitudinal, *Trans*. transverse, *BA* basal anterior, *BI* basal inferior, *BAS* basal anteroseptal, *BIL* basal inferolateral, *MA* mid anterior, *MI* mid inferior, *MAS* mid anteroseptal, *MII* mid inferolateral, *MAI* mid anterolateral, *MIS* mid inferoseptal, *ApA* apical anterior, *ApI* apical inferior, *ApL* apical lateral, *ApS* apical septal

The 2D-STE revealed that GLS (Fig 1a, b) was significantly improved as early as 1 day after CTO-PCI (Table 2).

These results indicated that 2D-STE could detect early subclinical LV systolic dysfunction in CTO patients after percutaneous revascularization.

## Discussion

This study found that the LVEF tended to improve at approximately 3 and 6 months after percutaneous revascularization in patients with CTO. Using 2D-STE, GLS was observed to be restored as early as 1 day after CTO-PCI. These results demonstrate that 2D-STE is a reliable way to monitor early subclinical LV changes.

Myocardium in the perfusion territory of a CTO can be functional, dysfunctional but viable, or dysfunctional and nonviable [14–16]. The targeted area of heart tissues in CTO patients comprise hibernating myocardium [17]. Biopsies of hibernating myocardium always show defects in nearly all cells [18]. The pathological changes include loss of sarcomeres and myofibrils in the center of the cells, absence of contractile material in the perinuclear areas, and presence of cellular debris in the enlarged extracellular space [19]. Vanoverschelde et al. found a relation between the extent of structural changes and the rate of recovery using myocardial biopsies [20]. The dysfunctional heart tissue in CTO patients can be improved after revascularization [21]. Steg and colleagues found that the recovery time of dysfunctional myocardium is dependent on the extent of damage at the cellular level, which is affected by different factors such as the duration and severity of ischemia [22]. However, more sensitive techniques are needed to assess the recovery of dysfunctional myocardium.

In this research, improvement of LVEF was observed for up to 3 and 6 months. However, by 2D-STE, the GLS improvement was observed as early as 1 day after CTO-PCI. 2D-STE is an automated and quantitative technique for the measurement of cardiac mechanics, which is based on the speckle interference of ultrasound beams within tissues. Speckles are tracked on a frame-by-frame basis throughout the cardiac cycle [23], which has more advantages than conventional echocardiography, including angle independency, free of tethering and translation effects, low signal-to-noise ratio, and low measurement variability [24, 25].

Systolic dysfunction might initially appear in the longitudinal direction, as the longitudinally oriented subendocardial fibers are more vulnerable to myocardial ischemia and fibrosis. GLS correlates well with EF measured by echocardiography, and GLS is a superior predictor of outcome compared with LVEF [26]. The EF measured by conventional echocardiography could show radial and partly longitudinal functions, whereas GLS could indicate longitudinal function.

### Limitations

There are several limitations in this study. There was a lack of control patients without intervention. Drug and other treatments may synergize the effects of revascularization on LV function. Only three time points were used in the follow-ups. More time points should be included to describe continuous improvement of heart function.

## Conclusion

PCI treatment can effectively improve LV function in patients with CTO. The results of this study provide evidence to support the clinical use of 2D-STE to monitor the early changes of LV function.
